# Decoding benign prostatic hyperplasia at single-cell resolution: heterogeneity, inflammation, and beyond androgen-driven pathogenesis

**DOI:** 10.3389/fimmu.2026.1881717

**Published:** 2026-07-24

**Authors:** Yihao Liao, Siyuan Jiang, Haohan Wang, Sihai Zhou, Honggang Yuan, Xuanxuan Zou, Xiaobo Chen

**Affiliations:** 1 Department of Urology, The First College of Clinical Medical Science, China Three Gorges University & Yichang Central People’s Hospital, Yichang, China; 2 Hubei Provincial Clinical Research Center for Parkinson’s Disease, Research Center for Translational Medicine, Xiangyang No.1 People’s Hospital, Hubei University of Medicine, Xiangyang, China

**Keywords:** benign prostatic hyperplasia, cellular senescence, immune microenvironment, precision medicine, single-cell RNA sequencing

## Abstract

Benign prostatic hyperplasia (BPH) represents a highly prevalent age-related disorder, traditionally managed through androgen-driven pathways. However, the limited efficacy of conventional hormonal therapies in a substantial subset of patients necessitates the investigation of alternative pathogenic mechanisms. The emergence of single-cell RNA sequencing (scRNA-seq) has provided the necessary resolution to map cellular heterogeneity and microenvironmental dynamics within the prostate. This review synthesizes recent advancements in applying scRNA-seq to BPH research, highlighting a transition from a homogeneous, hormone-centric view toward recognizing BPH as a complex, heterogeneous process driven by multifaceted cell-immune interactions. We detail how scRNA-seq has identified distinct cellular subsets within the hyperplastic transition zone, including novel basal epithelial subtypes and activated fibroblast populations that contribute directly to nodule formation and disease progression. Furthermore, we examine the central role of chronic inflammation, mediated by immune cell infiltration and senescence-associated secretory phenotypes (SASP), in perpetuating a proliferative microenvironment. The technology also clarifies mechanisms underlying treatment resistance and identifies potential biomarkers and novel therapeutic targets beyond the androgen axis, such as the CXCL13/CD4^+^T cell axis and granzyme K pathways. Looking forward, integrating scRNA-seq with spatial multi-omics aims to construct a comprehensive spatiotemporal atlas of BPH, facilitating molecular subtyping and the development of precision, microenvironment-targeted therapies. In conclusion, scRNA-seq is redefining the pathophysiological landscape of BPH, offering a path toward innovative, non-androgenic therapeutic strategies aimed at achieving true disease modification.

## 1 Introduction

Benign prostatic hyperplasia (BPH) is characterized by cellular hyperplasia and the formation of nodules within the prostatic transition zone, predominantly affecting middle-aged and elderly men (1–3). This condition is a primary driver of lower urinary tract symptoms (LUTS) due to bladder outlet obstruction (4). As the leading urological condition among aging men, BPH exhibits a prevalence rate of 50% to 75% in those aged 50 and older, increasing to 80% in men aged 70 and above (5, 6). It significantly compromises the quality of life in this population, with the associated symptom burden often exceeding that of other urological disorders. Despite its prevalence, the pathophysiological mechanisms underlying LUTS/BPH remain inadequately understood. It is generally accepted that BPH progression is mediated by androgen receptor-related signaling pathways (7–9). Current mainstream treatments include castration and androgen receptor-targeted therapies. However, a substantial proportion of patients are refractory to these treatments and ultimately require surgical intervention (10, 11). Clarifying the pathogenesis of androgen-independent prostate hyperplasia, particularly in cases unresponsive to hormonal blockade, has emerged as a focal point within this field (12). Decoding these novel mechanisms is essential for understanding the fundamental nature of the disease and identifying new therapeutic targets. Furthermore, this research establishes a crucial scientific foundation for the clinical development of more effective clinical strategies.

Traditional gene expression analysis techniques, such as real-time quantitative polymerase chain reaction (RT-qPCR) and bulk RNA sequencing, predominantly quantify average expression levels across bulk cell populations. These conventional approaches frequently mask intrinsic cellular heterogeneity, potentially overlooking the contributions of rare yet functional cell subsets to disease progression. The advent of single-cell RNA sequencing (scRNA-seq) technology has overcome these constraints by facilitating high-resolution analysis of the transcriptome, metabolome, and epigenome at single-cell resolution, thereby enabling an unbiased characterization of cellular heterogeneity (13–15). Moreover, through cellular trajectory analysis, scRNA-seq technology can reconstruct dynamic cell-state transitions and intercellular signaling networks during disease development (16, 17). Currently, scRNA-seq technology is extensively employed in the investigation of various diseases, including the cancer-related cellular microenvironment (CCMS) (14, 18). It provides comprehensive insights into cellular diversity across tissues, and disease models, elucidating the pivotal roles played by diverse cell subsets in disease evolution and contributing to numerous breakthroughs (19–21). However, the application of scRNA-seq in BPH research is still in its nascent stages. When synergized with traditional methodologies, scRNA-seq offers a more refined elucidation of the molecular mechanisms underlying BPH. This approach facilitates the identification and functional characterization of distinct cellular subsets, particularly the immune cell landscape within prostatic tissue. Such insights are anticipated to drive the development of innovative therapeutic strategies, particularly for refractory cases exhibiting poor responses to conventional medical treatments.

To better elucidate the application and advantages of scRNA-seq technology in BPH research, this review synthesizes the most recent findings derived from single-cell analyses. While existing reviews primarily focus on cataloging cellular atlases, they often lack a systematic integration of “non-androgen-driven mechanisms.” Consequently, the core clinical challenge of resistance to androgen-targeted therapies remains insufficiently addressed (22, 23). Furthermore, there is a paucity of critical synthesis regarding conflicting results from single-cell studies; discrepancies in fibroblast subpopulation functions, the causality of inflammation, and the role of AR-negative cells have yet to be reconciled through rigorous academic perspectives. Crucially, a clear translational framework for bridging laboratory findings with clinical practice remains elusive, resulting in a disconnect between molecular subtyping and clinical indicators. Centered on the theme “Beyond Androgen-Driven Pathways”, this review systematically organizes non-androgen-driven signaling elucidated by scRNA-seq, clarifying their pathogenic mechanisms independent of hormonal pathways. Concurrently, it critically reconciles conflicting results within the field and proposes integrated academic perspectives, aiming to establish a translational roadmap for clinical applications (**Table 1**). These findings will lay a robust foundation for advancing precision medicine in BPH and substantially expand the mechanistic understanding of non-AR related signaling pathways driven by specific cellular subpopulations.

**Table 1:** Application of single-cell sequencing technology in benign prostatic hyperplasia.

Sequencing subject	Cell subpopulation	Key factors	Signaling pathways	Results	Reference
BPH tissues	Fibroblasts	CSF1/CSF1R	PI3K/Aktsignaling	Fibroblasts promote the proliferation of BPH by secreting CSF1 and activating PI3K/Akt pathway	(83)
BPH tissues Network Pharmacology	NA	TGF-β1 IGF1 ITGA4	PI3K/Aktsignaling	Resveratrol significantly inhibited PI3K and Akt phosphorylation, pathway activation and BPH progression	(87)
BPH tissues	Epithelial cells	CXCL13	CXCR5/HLA-DR	CXCL13 enhances CD4+T cell infiltration and promotes clearance of prostate aging epithelial cells through CXCR5	(73)
Large- and small- volume BPH tissues	Fibroblasts	Granzyme K	SASP signaling	An age-associated CD8+ T cell subset (Taa) in large volume BPH secrete Granzyme K, which promotes the secretion of SASP cytokines in fibroblasts, then enhances CD8+T cell infiltration, exacerbating BPH hyperplasia	(5)
BPH tissuesmendelian randomization	Fibroblasts Macrophage T cell epithelial cells	ATM,TP53 ATRAID MAP2K1 ITPR1 SENP7	Senescence-related signaling (via forecast)	MAP2K1, ATM, ATRAID, and TP53 were identified as protective factors against BPH, whereas ITPR1 and SENP7 were associated with an increased risk of BPH	(95)
BPH treated with or without finasteride	Basal cells epithelial cells	TGF-β1	EGFR/PCNA signaling	Finasteride treatment leads to epithelial cell apoptosis, promotes TGF-β1 secretion, activates EGFR/PCNA, and promotes basal cell proliferation	(49)
Peripheral blood mononuclear cells	mononuclear cells	MIF TGF-β1	Notch signaling	Mononuclear cholesterol storage and Notch signaling enhancement in BPH, with a unique immune microenvironment	(72)
BPH tissues	Basal cells epithelial cells	HSPA1A	ERK/JNK signaling	HSPA1A promotes the proliferation of BPH by inhibiting the ERK/JNK pathway and suppressing cell apoptosis	(96)
Large- and small- volume BPH tissues	Macrophage (TREM2/ MARCO)	NA	Lipid metabolism signaling	The abundance of TREM2/MARCO dual high macrophage subpopulations is positively correlated with the BPH volume and IPSS score	(71)
BPH tissues	basal cell (BE5)	C-FOS	EMT signaling	The BE5 basal cell subset is both the initiating cell for the formation of proliferative nodules and the transitional cell during the transition from luminal epithelium to basal cells	(42)
Transitional zone and peripheral zone in BPH	Fibroblasts	NA	Stem-and Inflammation- related Pathways Notch signaling	Fibroblasts in the transition zone exhibit stronger transcriptional activity in immunity and proliferation, but lower levels of activity in androgen lineage related pathways promote infiltration of immune cells such as NK cells	(46)
Prostate tissues	Fibroblasts	SFRP/DKK1	Wnt signaling	The distribution of fibroblast subtypes varies in the matrix and glands, and the progression of BPH is mediated by the upregulation of key immune regulatory pathways through the secretion of cytokines	(43)
Primary prostatic epithelial cell culture	Basal cell	CCN6 CXCL5	NA	There is significant cellular heterogeneity in the immune microenvironment of the peripheral and transitional zones of the prostate, and the upregulation of characteristic genes is specific	(45)

## 2 Single-cell transcriptomic panoramic maps of the normal prostate and BPH

**Figure 1:**
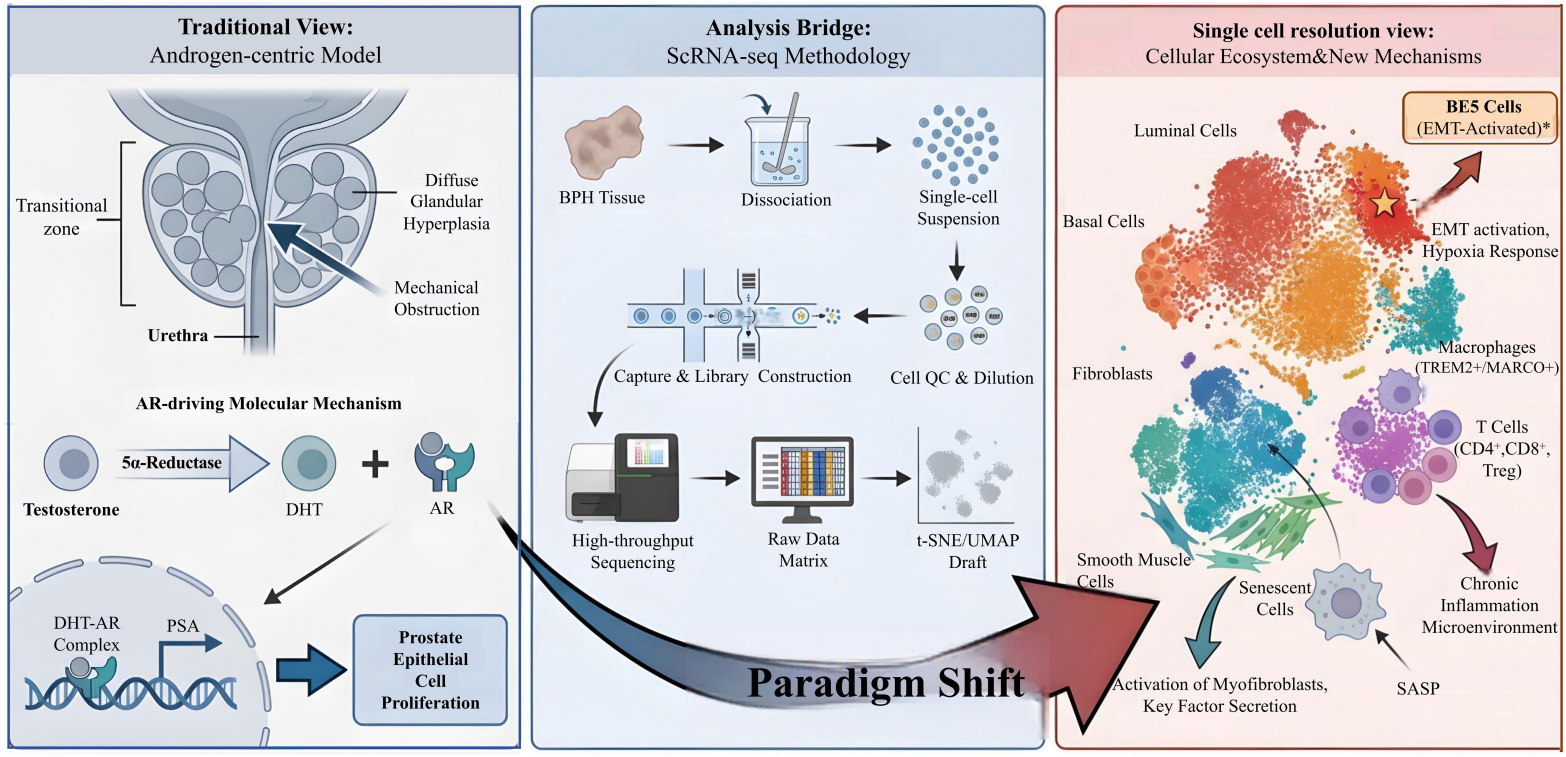
The paradigm shift in BPH: from an androgen-centric view to a cellular ecosystem. The traditional model (left) simplifies BPH pathogenesis to androgen-driven epithelial hyperplasia causing urethral obstruction: Testosterone is converted to dihydrotestosterone by 5α-reductase, Dihydrotestosterone (DHT) binds to androgen receptor Androgen Receptor (AR) and activates downstream proliferation related pathways, inducing excessive proliferation of prostate epithelial and stromal cells, which in turn leads to prostate hyperplasia. The central panel outlines the scRNA-seq workflow:BPH tissue dissociates into single-cell suspension, which undergoes quality control sorting, capture library construction, sequencing, and bioinformatics analysis to complete the entire single-cell sequencing process. The resulting single-cell map (right) reveals a heterogeneous cellular ecosystem in BPH, including epithelial cell (e.g., Luminal, Basal and BE5 cells), stromal (e.g., Fibroblasts, smooth Muscle Cell), and immune (e.g., Macrophages, CD4^+^, CD8^+^, Treg cells) populations, and highlights non-androgenic drivers such as chronic inflammation, senescence (SASP), and stromal activation, illustrating a fundamental shift in understanding. scRNA-seq, single-cell RNA sequencing; SASP, senescence-associated secretory phenotype.

Characterizing the heterogeneity of prostate cell subpopulations provides a foundational framework for understanding the non-androgen-driven mechanisms underlying BPH. scRNA-seq enables a transformative shift from the traditional androgen-centric model toward a high-resolution view of the cellular ecosystem (**Figure 1**). Based on single-cell sequencing data, this chapter analyzes cellular dynamics and regional reconfiguration—particularly within the transition zone—focusing on identifying key pathogenic subsets such as epithelial-mesenchymal transition(EMT)-activated BE5 cells and senescent cells harboring a secretory phenotype (SASP). These findings delineate a complex cellular landscape supporting the central hypotheses of this review, where microenvironmental remodeling, spatial heterogeneity, and cellular evolution drive BPH progression independently of androgen signaling (**Figure 1**).

### 2.1 Cell types and functions in the normal prostate

Prostatic homeostasis is maintained by the coordinated secretory function of luminal cells, the regenerative capacity of basal cells, the precise regulation of neuroendocrine cells, the structural support and signal transduction provided by stromal cells, and the balance between immune defense and immune tolerance (24, 25). This complex cellular ecosystem collectively supports the prostate’s roles in secretion, excretion, and reproductive protection. Dysregulation of cellular composition or intercellular communication underscores the pathogenesis of diseases such as BPH or prostate cancer. Consequently, the prostate can be categorized into three major compartments: the epithelium, the stroma, and the immune microenvironment (26).

The prostate epithelium is a highly organized glandular structure primarily composed of three cell types arranged in a pseudostratified bilayer: luminal cells in the inner layer, basal cells in the outer layer, and neuroendocrine cells sparsely distributed between them (27, 28). The basal layer, adjacent to the basement membrane, harbors stem cell niches with the capacity for self-renewal and multilineage differentiation into luminal and neuroendocrine lineages. These cells are crucial for maintaining epithelial homeostasis and promoting tissue repair (29). Luminal cells form the secretory epithelium lining the glandular lumen (30). They are responsible for synthesizing and secreting key components of prostatic fluid, including prostate-specific antigen (PSA), prostatic acid phosphatase (PAP), zinc, citrate, and spermidine (25). These components are essential for semen liquefaction, sperm motility, and antimicrobial defense. Neuroendocrine cells, conversely, regulate the growth, differentiation, and secretory functions of adjacent epithelial cells through paracrine signaling by releasing bioactive peptides and amines, such as serotonin, chromogranin A, calcitonin gene-related peptide, and growth hormone-releasing hormone (31). This regulatory function constitutes a key interface between the autonomic nervous system and the prostate epithelium.

The stromal compartment constitutes the supportive microenvironment for epithelial cells. Characterized by an abundant extracellular matrix (ECM), stromal cells orchestrate epithelial development, differentiation, and homeostatic maintenance, primarily comprising fibroblasts, smooth muscle cells, and endothelial cells (32). Fibroblasts facilitate structural scaffolding and tissue repair by synthesizing ECM components, while simultaneously modulating epithelial cell function by secreting various growth factors, including FGFs, TGF-β, IGF-1, and Wnts. Smooth muscle cells comprise the fibromuscular stroma and the dense prostatic capsule; their rhythmic contractions facilitate the propulsion of prostatic fluid into the urethra during ejaculation (33). Endothelial cells form the vascular endothelium, modulating prostate function and driving angiogenesis.

The normal prostate harbors a dynamic immune microenvironment, evolved to preserve tissue homeostasis, provide surveillance, and maintain tolerance toward self-antigens (such as PSA) (34, 35). Immune cell populations are primarily categorized into resident subsets (including resident macrophages, T lymphocytes, dendritic cells, innate lymphoid cells, and natural killer cells) and infiltrating subsets (including neutrophils, plasma cells, and activated effector T cells) (36, 37). While resident cells are predominantly responsible for tissue remodeling, clearance of apoptotic cells, maintenance of immune tolerance, and immune surveillance, infiltrating subsets typically associated with subclinical injury, infection, or inflammation. Furthermore, the distribution, diversity, and proportions of these cells within the prostate are highly contingent upon the organ’s physiological and pathological state.

A comprehensive understanding of BPH pathogenesis necessitates establishing its physiological baseline. The diverse cellular array and the intricate, dynamic immune landscape within the prostate collectively maintain normal function; any disruption in cellular composition or signaling will lead to homeostatic imbalance. Crucially, an expansion of AR-negative cell populations may drive the development of androgen-independent BPH.

### 2.2 Cell subpopulation reconfiguration and heterogeneity in BPH

Anatomically, the prostate is divided into four distinct anatomical zones: the transition zone (TZ), the peripheral zone (PZ), the central zone (CZ), and the periurethral glandular zone—the latter being the smallest in volume (38). The TZ surrounds the proximal urethra and lies anterior to the central zone, comprising approximately 5%-10% of the total prostate volume; it is the predominant site of BPH development. With advancing age, hyperplastic expansion in this region encroaches upon the urethra, leading to obstructive voiding symptoms. The peripheral zone is located posterolateral to the prostate; it is the largest region, representing 70%-75% of the gland (39, 40). This region is frequently associated with prostate cancer and chronic prostatitis and is the area palpable during digital rectal examination (41). Therefore, investigating the cellular composition and immune microenvironment of the TZ is pivotal to elucidating the pathophysiological mechanisms of BPH. Characterizing alterations in the epithelial-to-stromal ratio and identifying abnormally activated cell populations can help identify key drivers of the disease. These insights are crucial for advancing diagnostic and therapeutic strategies for BPH.

Xiawei Fei et al. confirmed that hyperplastic nodules are the primary drivers of prostatic enlargement and LUTS in BPH patients (42). Using scRNA-seq, the researchers identified a novel basal epithelial subtype, designated as BE5. which serves as both the primordial source of nodule formation and a critical intermediate state within the luminal-to-basal differentiation trajectory. BE5 cells display a rod-like morphology, an enhanced hypoxic response, and upregulated C-FOS expression; these features collectively trigger the EMT pathway, thereby driving BPH pathogenesis. Consequently, targeting BE5 populations or pharmacologically inhibiting C-FOS signaling may attenuate BPH progression and alleviate associated LUTS. Similarly, Diya B Joseph et al. utilized scRNA-seq to categorize three smooth muscle cell subtypes (prostatic smooth muscle cells, pericellular cells, and vascular smooth muscle cells) and two fibroblast subtypes (periepithelial fibroblasts and stromal fibroblasts) within the prostate (43). By elucidating the distinct spatial distribution of three fibroblast subsets across the stromal and glandular compartments, the study revealed their functional diversity in disease progression—specifically through the secretion of growth factors, morphogens, and chemokines, as well as their engagement in key immunoregulatory pathways. Based on these mechanistic insights, the authors suggest that modulating the secretion of these regulatory factors or reconfiguring the stromal cellular landscape may represent a potent intervention strategy for BPH.

Given that the TZ and PZ are the primary sites for BPH and prostate cancer, respectively, elucidating the divergent cellular landscapes and immune microenvironments between these regions provides critical insights into the BPH homeostatic milieu through the “soil–seed” perspective. This analytical framework may help target the microenvironmental drivers of BPH pathogenesis and facilitate innovative therapeutic interventions (44). Jordan E. Vellky et al. performed scRNA-seq on primary cultured prostate epithelial cells, identifying distinct cellular signatures between the PZ and TZ. Notably, basal epithelial cells in the TZ exert a pivotal influence on BPH development. Compared to the central and peripheral zones, CCN6 is specifically enriched in TZ basal epithelial cells, while CXCL5 is significantly elevated in the TZ stroma (45). These findings establish a mechanistic link between BPH progression and the orchestration of local immune responses. Qiuxia Yan et al. conducted a comparative scRNA-seq analysis of the transition and peripheral zones in elderly BPH patients and identified a pronounced expansion of the fibroblast compartment in the TZ. These cells potentiate the Notch signaling pathway through cytokine secretion, thereby activating stemness and inflammation-related pathways while exhibiting diminished androgenic signaling (46). Immunologically, TZ microenvironment is highly heterogeneous and skewed, featuring increased infiltration of NK cells and regulatory T cells (Tregs). CD8^+^ T cells and macrophages display a bifurcated inflammatory profile, encompassing both activation and suppression phenotypes. Regarding stromal components, the TZ exhibits a higher fibroblast density with robust transcriptomic activity related to immunity and proliferation. These findings suggest that the TZ’s heightened susceptibility to BPH is intrinsically linked to its unique cellular heterogeneity and immune landscape (5). Interventions targeting specific cellular subsets or modulating the TZ-specific immune milieu may inhibit prostatic hyperplasia by restoring local homeostatic balance.

Although scRNA-seq has significantly advanced our understanding of the cellular landscape in BPH, certain findings remain a subject of active debate. Regarding AR-negative luminal cells, the discrepancies in existing research—specifically whether they function as the primary drivers of BPH—stem from regional heterogeneity (TZ vs. PZ) and disease staging (46–48). Single-cell sequencing has confirmed that AR-negative luminal cells might be primarily enriched within hyperplastic nodules in the transitional zone; their proliferation operates independently of AR signaling, making them a critical subpopulation in non-androgen-driven BPH (49). Future studies necessitate the validation of their stemness potential via lineage tracing. Concurrently, a consensus on the nomenclature and functional characterization of fibroblast subpopulations has yet to be reached. The fundamental cause is the lack of standardized marker genes, functional validation criteria, and hierarchical classification systems. Given the high heterogeneity of scRNA-seq datasets, a unified annotation workflow must be establishing a unified annotation workflow is essential to integrate these findings, while spatial transcriptomics should be employed to delineate their spatial architecture and pathogenic roles. Currently, the primary challenges in scRNA-seq-based BPH research include limited sample sizes and the paucity of large-scale, multicenter cohorts; these issues warrant further investigation. To bridge the disparate nomenclatures observed across independent studies—such as periepithelial fibroblasts, stromal fibroblasts, and myofibroblasts—current scRNA-seq analyses increasingly employ classic stromal marker combinations as molecular anchors. Specifically, PDGFRα (platelet-derived growth factor receptor α) has been confirmed to be expressed in BPH stromal cells (50); PDGFRβ and the extracellular matrix component COL1A1 are transcriptionally regulated in prostatic fibroblasts (51). FAP (fibroblast activation protein) is recognized as a marker of activated stromal cells (52), and is expressed in benign prostatic tissues including proliferative inflammatory atrophy (PIA) (53). Additionally, ACTA2/α-SMA (alpha-smooth muscle actin), along with COL1A1 and COL3A1, exhibits upregulated expression in primary prostatic fibroblasts derived from BPH tissues, reflecting the contractile and matrix-remodeling phenotypes of pathogenic fibroblast subsets. This marker−based framework enables the translation of diverse naming systems into a broadly unified classification stratified by spatial niche, contractile properties, and inflammatory secretory profiles, thereby providing a conceptual basis for cross−dataset comparisons and functional interpretation within the BPH microenvironment.

### 2.3 New insights into the pathogenesis of BPH: accumulation of senescent cells

Recent studies have revealed that BPH is not merely a disorder of glandular “overgrowth”, but rather a chronic inflammatory proliferative disease closely associated with organ aging and driven by the accumulation of senescent cells and their pro-inflammatory secretome (54–57). Senescent epithelial cells and their SASP constitute a central mechanism that initiates and sustains this pathological cycle. With advancing age, prostate epithelial cells (particularly acinar and ductal epithelial cells) gradually accrue cellular damage due to prolonged exposure to oxidative stress (e.g., from metabolites or mild infections) and hormonal fluctuations, ultimately transitioning into a senescent state. Owing to age-related declines in immune clearance function, these senescent cells evade effective clearance and progressively accumulate within the prostatic tissue (58, 59). The accumulating senescent epithelial cells persistently secrete SASP factors, instigating a cascade of local microenvironmental shifts (60). This establishes a chronic inflammatory milieu that stimulates stromal and epithelial cell proliferation, fibrosis, and angiogenesis, ultimately leading to expansion of both glandular and stromal compartments. The resultant increase in prostate volume compresses the urethra, manifesting as lower urinary tract symptoms. Within this process, androgens act primarily as “permissive factors” or “amplifiers,” providing the requisite physiological background for prostate cell growth while sensitizing cells to SASP-derived mitogenic signals (61–63). This contemporary understanding is shifting the therapeutic focus from conventional “gland shrinkage” or “muscle relaxation” toward fundamental strategies targeting cellular senescence. Through scRNA-seq analysis, Zheng Li et al. have identified CXCL13 as a pivotal chemokine secreted by senescent prostate epithelial cells. CXCL13 recruits CD4^+^T cells via the CXCR5 receptor, thereby facilitating the immune recognition and clearance of senescent cells through HLA-DR mediated mechanisms. Specifically, CD4^+^ cytotoxic T lymphocytes (CTLs) are recruited to mediate senescent cell clearance, whereas Tregs act to suppress this immunosurveillance. In a testosterone-induced BPH mouse model, exogenous CXCL13 administration augmented CD4+ T cell infiltration and attenuated epithelial senescence; conversely, CD4+ T cells depletion abolished these protective effects. These findings underscore the critical role of the CXCL13/CXCR5/HLA-DR/CD4^+^T cell axis in the clearance of senescent cells in BPH (64). Collectively, this work unveils a critical immune-surveillance mechanism and suggests that targeting the CXCL13/CD4^+^T cell axis may offer a novel therapeutic strategy. By integrating scRNA-seq data datasets of BPH-isolated immune cells, Meaghan M Broman et al. demonstrated that granzyme K-mediated(GZMK) stimulation the release of SASP-related cytokines from prostate stromal fibroblasts promoting the recruitment and activation of CD8^+^T cells (5). This process subsequently drives the localized accumulation of senescent cells.

Therapeutic strategies targeting senescent cells offer a novel direction for treating refractory BPH resistant to androgen-targeted therapies, driving a shift in the BPH treatment paradigm from traditional symptomatic relief toward etiological intervention. Based on scRNA-seq findings, current core therapeutic strategies fall into three categories: First, senolytic-mediated specific clearance of senescent cells, which selectively eliminates senescent prostate epithelial cells and abrogates the sustained secretion of SASP; preclinical models have demonstrated that this approach can reverse prostate fibrosis and glandular hyperplasia; Second, interventions targeting the senescence-immune surveillance axis, including modulating the CXCL13/CXCR5 axis to enhance the immune clearance of senescent cells and targeting granzyme K to inhibit aberrant SASP secretion, have both demonstrated significant efficacy in animal models; Third, targeted inhibition of the SASP signaling pathway, which blocks the senescence-SASP-inflammation cascade to suppress androgen-independent stromal fibrosis and epithelial proliferation.

In summary, within the context of BPH, senescent epithelial cells have transitioned from their previously overlooked role as “bystanders” to become pivotal “active drivers” of disease pathogenesis. These therapeutic strategies can complement existing mainstream treatment regimens and are particularly suitable for refractory, large-volume inflammatory BPH cases that remain unresponsive to androgen therapy. Although most relevant studies remain in the preclinical or early clinical stages, the selective clearance of senescent cells and targeted inhibition of SASP offer promising new avenues for BPH treatment with significant translational potential.

## 3 Microenvironmental landscapes of BPH at single-cell resolution

**Figure 2:**
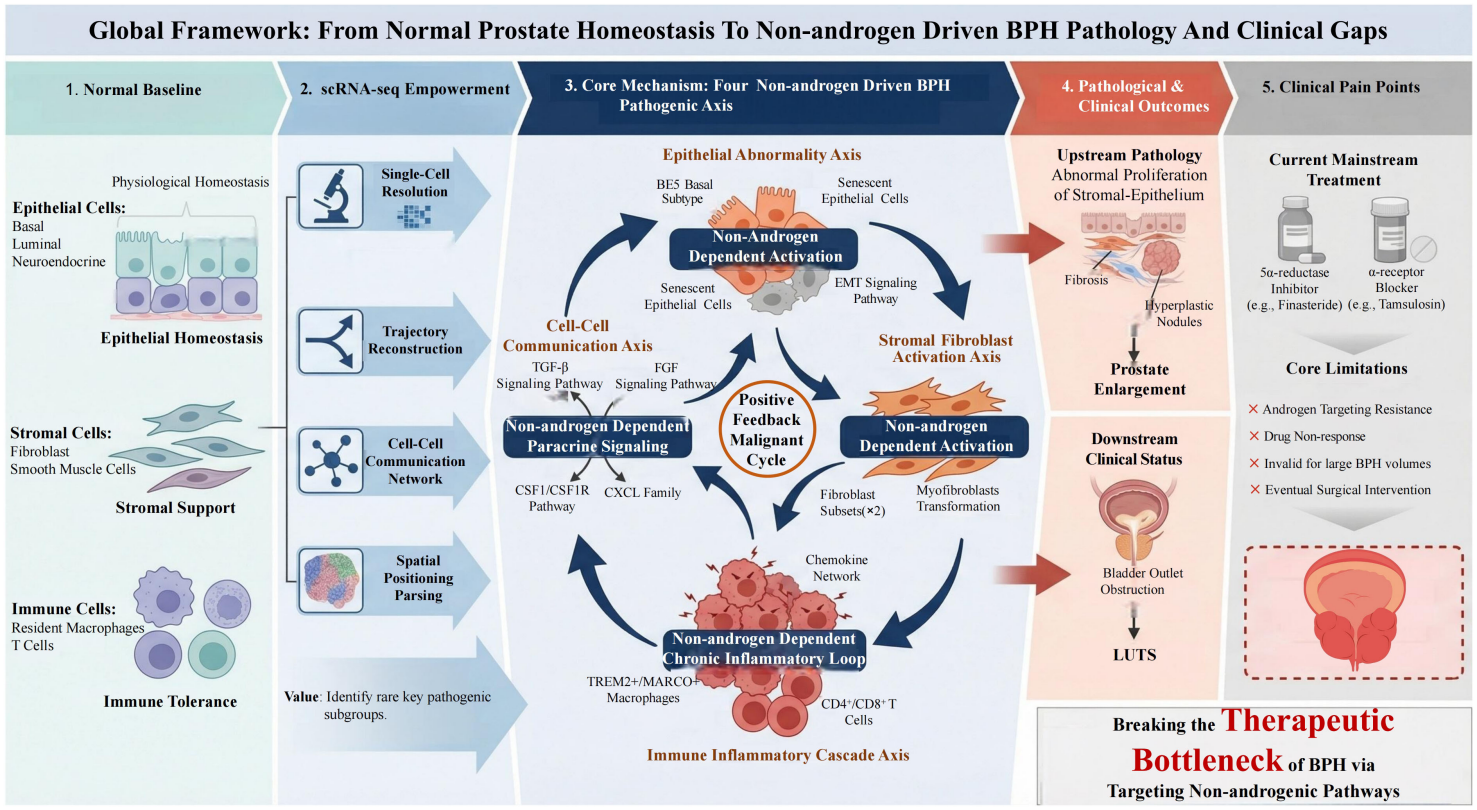
Global framework: from normal prostate homeostasis to non-androgen driven BPH pathology and clinical gaps. This illustration delineates the continuum from normal prostate homeostasis to benign prostatic hyperplasia (BPH) pathology, anchored by single-cell RNA sequencing (scRNA-seq) technologies that deconvolute cellular heterogeneity and intercellular communication. At the core are four non-androgen dependent pathogenic axes—epithelial abnormality, stromal fibroblast activation, immune inflammation, and paracrine signaling networks—forming a central positive feedback loop that drives disease progression independent of androgen signaling. These mechanisms culminate in pathological fibrosis, nodule formation, and clinical manifestations such as bladder outlet obstruction and lower urinary tract symptoms, ultimately exposing the critical gap in current androgen-targeted therapies. By highlighting these non-androgen driven pathways, the framework underscores a paradigm shift toward disease-modifying strategies to overcome the therapeutic bottlenecks in BPH management.

The chronic inflammatory microenvironment and aberrant intercellular communication constitute the central hub of the non-androgen-driven mechanisms in BPH, potentially underpinning the resistance to androgen-targeted therapies (65–67).Single-cell resolution allows for the systematic parsing of a “central malignant loop” involving epithelial remodeling, pathological fibroblast activation, and immune-inflammatory cascades (**Figure 2**). Leveraging these insights, this chapter elucidates how distinct cell states—such as TREM2+/MARCO+ macrophages and myofibroblasts—reprogram the local milieu through paracrine networks (e.g., TGF-β, FGF, and CXCL families). Furthermore, we discuss how spatial transcriptomics has identified localized signaling circuits within hyperplastic nodules that operate independently of androgen signaling. These non-androgenic axes address core clinical unmet needs, providing a biological basis for therapeutic failure and eventual surgical intervention in refractory BPH (**Figure 2**).

### 3.1 Single-cell RNA sequencing reveals the immune infiltration landscape in BPH

The prostate immune microenvironment constitutes a complex network of resident and infiltrating immune cells that orchestrates the pathophysiological progression of BPH. Multiple studies have confirmed a positive correlation between prostate enlargement (PE) and the increased severity of histological inflammation. Under physiological conditions, these cells collectively maintain tissue homeostasis and immune tolerance. However, in pathological states, they shift from immune surveillance to driving chronic inflammation (36, 68). Through complex cytokine networks, they directly promote aberrant cell proliferation, tissue remodeling, and fibrosis within the prostate. These immune cells represent key mediators linking aging and hormonal backgrounds to clinical manifestations such as LUTS (37). A deeper understanding of how to modulate this cellular network within the immune microenvironment is crucial for developing novel immunotherapeutic strategies for the precision treatment of BPH.

Resident immune cells situated in the prostate stroma constitute the first line of immune surveillance. In BPH, they are activated by persistent endogenous and exogenous signals (69). Prostate-resident macrophages primarily exhibit an M2 phenotype (anti-inflammatory/repairing) and maintain tissue homeostasis under homeostatic conditions. In the chronic inflammatory milieu of BPH, these macrophages polarize toward pro-inflammatory M1-type cells and continuously secrete inflammatory mediators, promoting the proliferation of epithelial and stromal cells (70). Notably, scRNA-seq analysis has identified two distinct macrophage subpopulations in BPH—TREM2+ and MARCO+ macrophages—which are associated with reprogrammed lipid metabolism signaling pathways (71). Interestingly, classic M1/M2 polarization is not distinctly observed in BPH. Lipid-laden macrophages may exacerbate LUTS, particularly in patients with large-volume BPH. The abundance of TREM2+ and MARCO+ macrophages correlates positively with body mass index and symptom scores, and these non-polarized macrophages preferentially accumulate in larger prostates. Furthermore, scRNA-seq analysis of peripheral blood mononuclear cells (PBMCs) from BPH patients highlights the pivotal regulatory role of monocytes in immune modulation. Enhanced cholesterol storage and Notch signaling in monocytes may exert significant regulatory effects via the MIF/galectin/TGF-β signaling axis (72). The unique immune microenvironment and transcriptional heterogeneity in BPH provide potential biomarkers for distinguishing BPH subtypes and elucidating their underlying pathologenesis.

Meaghan M. Broman et al. revealed that fibroblasts in BPH promote the recruitment and activation of immune cells through the secretion of chemokines, leading to age-associated CD8^+^ T-cell infiltration into BPH tissue. The extent of this infiltration is positively correlated with the International Prostate Symptom Score (IPSS) (5). Furthermore, studies have found that CXCL13 secreted by prostate epithelial cells enhances CD4^+^ T cell infiltration in BPH, promotes immune recognition via HLA-DR, and thereby facilitates the clearance of senescent epithelial cells. This clearance is mediated by CD4^+^ cytotoxic T lymphocytes (CTLs) and antagonized by Tregs. Therefore, the composition and density of immune cells within the tissue (such as macrophages and CD4^+^ T cells) may serve as novel biomarkers for predicting BPH progression or treatment response; however, this warrants further investigation of additional immune cell subsets (64).

Regarding whether inflammation is a driving factor in the development of BPH or merely a concomitant phenomenon that arises after its onset, the findings mentioned above remain a subject of active in current BPH research. Collectively, the scRNA-seq data accumulated to date strongly suggest that chronic inflammation functions as a primary driving factor in the pathogenesis of BPH, rather than solely a secondary consequence following its onset. Multiple lines of evidence support this interpretation: single-cell trajectory analysis indicates that immune cell infiltration precedes the emergence of epithelial and stromal proliferation phenotypes (5, 73); clinical cohorts demonstrate a positive correlation between histological inflammation and BPH progression (74, 75), while mechanistic and clinical evidence suggests that inflammation may also contribute to therapeutic resistance in a subset of patients (76, 77); and anti-inflammatory treatment has been shown to significantly alleviate symptoms and glandular hyperplasia in patients with inflammatory BPH (78). Furthermore, proliferating BPH cells can recruit inflammatory cells via chemokines; the subsequent exacerbation of inflammation further propels BPH progression, thereby forming a positive feedback loop that accelerates disease development (79). These findings collectively indicate that chronic inflammation is a core driver of BPH onset, progression, and therapeutic resistance, constituting a key component of the non-androgen-driven mechanisms underlying BPH. This provides new therapeutic directions for BPH patients, particularly those who respond poorly to conventional therapies. Nevertheless, it is important to acknowledge that scRNA-seq of resected human tissues inherently captures static, end-stage snapshots of the disease process. Therefore, while the current data strongly implicate inflammation as an initiating event, definitive decoupling of the temporal sequence between inflammation and proliferation—and thus formal establishment of causality—will require future integration of temporal lineage-tracing studies or dynamic animal models that can track the evolution of these cellular events over time.

### 3.2 Activated fibroblasts as a core driving factor in the evolution of BPH

Fibroblasts within the prostate are not merely passive structural cells. During the pathophysiological progression of BPH, they undergo pathological activation and differentiate into myofibroblasts under the influence of multifaceted factors, including genetic susceptibility, age-related hormonal shifts (such as an increased estrogen-to-androgen ratio), chronic inflammation, and mechanical stress (80, 81). These activated fibroblasts are not only the primary cellular drivers of stromal hyperplasia and capable of directly forming hyperplastic nodules, but they also recruit inflammatory cells through the secretion of chemokines, thereby amplifying the inflammatory response. Furthermore, they promote aberrant epithelial proliferation and drive fibrotic remodeling of the microenvironment, creating a “fertile ground” for sustained proliferation and leading to impaired tissue elasticity. Zhan et al. have demonstrated that fibroblasts in BPH activate the PI3K/AKT signaling pathway by secreting CSF1, thereby promoting epithelial cell proliferation (82). Furthermore, T cell in large-volume BPH tissue secrete granzyme K, GZMK variably induces SASP (senescence-associated secretory phenotype) associated cytokine expression in prostate fibroblasts *in vitro*, suggesting that Taa cells may promote prostate cellular expansion and immune cell recruitment through GZMK-induced fibroblast SASP production, thereby promoting CD8^+^T cell infiltration and ultimately exacerbating clinical obstructive symptoms (5).

Matrix-epithelial interactions have been shown to be critical for prostate morphogenesis, differentiation, and the maintenance of homeostasis. Analysis of intercellular receptor-ligand interactions highlights the central role of fibroblasts in transmitting signals from growth factors, morphogens, and chemokines to endothelial cells, epithelial cells, and immune cells, thereby promoting immune cell infiltration. Notably, periepithelial fibroblasts express secreted Wnt inhibitors, such as SFRP and DKK1, which buffer stromal Wnt ligands and help establish a localized signaling microenvironment around individual prostate acini (43). Regional differences in fibroblast density between the proximal and distal regions of the prostate further modulate stromal signaling, with higher signaling activity observed around the proximal prostatic ducts. Consequently, contemporary BPH research has identified the targeted inhibition of fibroblast activation and associated signaling pathways (such as TGF-β signaling and mechanosensory pathways) as a promising therapeutic strategy. This approach aims to intervene in core pathogenic mechanisms at their source, rather than merely alleviating symptoms. Therefore, understanding fibroblast function is crucial for elucidating the complete pathological cascade underlying the progression of BPH from benign tissue overgrowth to clinical symptoms.

### 3.3 Intercellular communication networks in the BPH microenvironment at single-cell resolution

Based on single-cell sequencing data, this section elucidates two key non-androgen driven mechanisms in the BPH microenvironment: the infiltration patterns of immune cells and the pathological activation of fibroblasts. Intercellular communication networks serve as the central hub connecting these distinct cell subsets to form a non-androgen-dependent positive feedback loop. Traditional bulk sequencing only captures average gene expression at the tissue level and cannot resolve cell-type-specific or cell-state-specific *in situ* interactions; however, scRNA-seq and complementary spatial transcriptomics technologies have, for the first time, enabled single-cell resolution analysis of intercellular signaling in the BPH microenvironment. Crucially, these technologies have revealed numerous core paracrine signaling circuits that are independent of androgen pathways and drive disease progression and treatment resistance. This section systematically reviews research advances in BPH intercellular communication enabled by single-cell technologies, focusing on non-androgen-dependent core pathways, spatially specific interactions, and cell-state-specific dialogues, thereby providing theoretical support for non-androgenic therapeutic strategies targeting intercellular communication.

#### 3.3.1 The link between intercellular communication and non-androgen-driven mechanisms of BPH

Intercellular communication analysis using scRNA-seq provides a systematic approach for evaluating signaling networks between cells in the tissue microenvironment at single-cell resolution, leveraging ligand-receptor interactions. Compared to traditional methods, it overcomes key limitations such as the inability to determine the cellular origin of ligands and receptors, the difficulty in identifying interactions involving rare cell subpopulations, and the challenge of distinguishing between physiological and pathological signals. Consequently, scRNA-seq-based studies of intercellular communication facilitate the unbiased identification of cell-specific pathogenic pathways that operate independently of AR signaling.

In the TGF-β signaling pathway, activated fibroblasts and senescent epithelial cells serve as primary ligand-donor cells. The TGF-β ligands they secrete interact specifically with their cognate receptors on fibroblasts, epithelial cells, and immune cells to drive intercellular communication and mediate pathological activation of the pathway. ScRNA-seq confirmed that ligand-receptor pairs in this pathway are significantly enriched in the hyperplastic zone of the transition zone in BPH patients; furthermore, its activation is independent of AR transcriptional activity, as it continues to drive prostate fibrosis and hyperplasia in castrated mouse models. Notably, the TGF-β pathway is also significantly upregulated in patients refractory to finasteride treatment (43, 72). Ultimately, the aberrant activation of the TGF-β pathway drives the transformation of fibroblasts into myofibroblasts, extracellular matrix fibrosis, and androgen-independent proliferation of epithelial cells, establishing it as a core regulatory pathway in the progression of fibrotic BPH.

In the FGF (fibroblast growth factor) signaling pathway, periepithelial fibroblasts and stromal fibroblasts serve as key ligand-secreting cells; the FGF ligands they release interact specifically with their cognate receptors on basal epithelial cells (particularly the BE5 subtype) and luminal epithelial cells (42). ScRNA-seq confirms that the ligand-receptor pairs of this pathway are specifically enriched at the fibroblast-epithelial interface within hyperplastic nodules of the transition zone. Furthermore, the activation of the FGF pathway in BPH patients is independent of AR expression levels; in AR-knockdown epithelial cells, FGF still significantly promotes cell proliferation. Thus, Fibroblast-secreted ligands trigger aberrant FGF signaling, driving dysregulated proliferation of epithelial stem cells and the EMT process, thereby constituting the core paracrine mechanism for the formation of hyperplastic nodules (46).

In the Wnt signaling pathway, periepithelial fibroblasts and basal epithelial cells secrete Wnt inhibitors such as SFRP and DKK1, which act on prostate stem cells and basal epithelial cells via ligand-receptor interactions. ScRNA-seq data confirm that periepithelial fibroblasts establish a localized signaling microenvironment around the prostatic acini via these Wnt inhibitors, rendering Wnt pathway regulation independent of androgen levels and maintaining the proliferative potential of epithelial stem cells even under castration conditions (43). Aberrant activation of the Wnt pathway drives excessive proliferation of epithelial stem cells, disrupts epithelial homeostasis, and promotes the formation of hyperplastic nodules.

In the CSF1/CSF1R signaling pathway, activated fibroblasts secrete CSF1, which acts its target cells—macrophages and luminal epithelial cells—to activate downstream signaling. ScRNA-seq confirmed that the co-expression levels of CSF1 and CSF1R in BPH tissue positively correlate with prostate volume and IPSS scores. Furthermore, finasteride treatment does not affect CSF1 secretion by fibroblasts, and CSF1/CSF1R pathway inhibitors can suppress BPH progression in castrated mouse models. Aberrant activation of the CSF1/CSF1R axis recruits and polarizes macrophages into a pro-inflammatory phenotype, directly driving epithelial cell proliferation and amplifying the positive feedback loop between chronic inflammation and proliferation (82).

Within the CXCL chemokine family signaling pathway, senescent epithelial cells and activated fibroblasts recruit immune cells such as CD4^+^/CD8^+^ T cells, macrophages, and NK cells, by secreting specific chemokines. ScRNA-seq analysis confirmed that specific CXCL/CXCR pairs, such as CXCL13/CXCR5 and CXCL5/CXCR2, are enriched in the inflammatory regions of BPH. Furthermore, the secretion of CXCL13 by senescent epithelial cells is independent of AR signaling, and androgen deprivation therapy does not affect the immune cell recruitment and infiltration mediated by this pathway (64, 83). Consequently, CXCL chemokine-mediated immune cell recruitment and infiltration amplify chronic inflammatory cascades, promoting both the immune surveillance and immune evasion of senescent cells, thereby constituting a core regulatory pathway in inflammatory BPH.

By elucidating the aforementioned core non-androgenic regulatory pathways and mechanisms in BPH, it is clear that activated fibroblasts serve as the central hub of the BPH intercellular communication network, while epithelial cells act as the primary effectors for signal reception and proliferation, and immune cells represent the critical link in the amplification of the inflammatory cascade. Together, these three components form pathogenic positive feedback loop through the aforementioned non-androgen-dependent pathways.

#### 3.3.2 Intercellular interactions within hyperplastic nodules

Conventional scRNA-seq requires dissociating tissue into a single-cell suspensions, which leads to the loss of *in situ* spatial information and precludes distinguish between systemic tissue-level interactions and localized interactions within hyperplastic nodules. In contrast, integrating single-cell sequencing with spatial transcriptomics enables both precise cell-type annotation and spatial mapping, allowing for the clearer resolution of local signaling loops within BPH nodules. Elucidating the specific enrichment of non-androgenic pathways within these nodules provides the structural basis for resistance to androgen-targeted therapies. Based on joint analysis using these multimodal technologies, the spatial architecture of BPH hyperplastic nodules has been characterized: the core of the nodule consists of clonally proliferating epithelial cells (enriched in BE5 basal cells) and abundant activated fibroblasts; surrounding the nodule are focally infiltrating immune cells (T cells, macrophages, etc.) and angiogenic vessels. At the outermost periphery lies relatively normal prostate tissue. Furthermore, AR expression levels within the nodules are significantly lower than in the surrounding normal tissue, whereas ligand-receptor pairs from non-androgenic pathways are significantly enriched, indicating that nodular hyperplasia in BPH is not primarily regulated by the AR signaling pathway.

Periepithelial fibroblasts adjacent to the glands within nodules exhibit focal, high levels of FGF and TGF-β secretion, which directly act on nearby BE5 basal cells and luminal epithelial cells (42), driving non-androgen-dependent epithelial proliferation and forming a localized positive feedback loop of “fibroblast activation → epithelial proliferation → nodule enlargement.” This ligand-receptor enrichment is confined to the fibroblast-epithelial interface within the nodule and is not observed in adjacent normal tissue; furthermore, AR expression levels within the nodule do not correlate with the degree of activation of pathway activation, and finasteride treatment fails to disrupt this circuitry (44, 49).

Focal clusters of senescent epithelial cells within nodules highly secrete chemokines such as CXCL13, recruiting CD4^+^ T cells and Tregs *in situ* to form a localized immune microenvironment; The immunosuppressive effect of Tregs prevents the clearance of senescent cells, promoting their accumulation and SASP secretion, thereby amplifying local inflammation (64). Senescent cells and T cells exhibit significant colocalization within nodules but not in non-proliferative regions, suggesting a locally specific interaction between the two within nodules. Furthermore, the secretion of chemokines by senescent cells is independent of AR signaling, and androgen deprivation therapy fails to reverse this local inflammatory loop (5, 44).

These findings collectively indicate that within the proliferative nodules of the prostate transition zone, non-androgen-dependent local interactions are significantly more active than in the surrounding transition zone tissue, and that transition zone fibroblasts are more prone to forming localized signaling loops. From the perspective of spatial interactions, this explains why the transition zone represents the primary site of BPH development.

#### 3.3.3 Limitations of current research

Although scRNA-seq and spatial transcriptomics have significantly advanced our understanding of BPH intercellular communication networks and revealed various non-androgen-dependent pathogenic pathways, existing studies still face fundamental limitations across multiple levels, impeding the translation of research findings into clinical practice. Most studies remain at the level of bioinformatics predictions, with a notable lack of rigorous *in vivo* and *in vitro* functional validation—this remains the most critical limitation in the field. Furthermore, nearly all existing studies are based on surgically resected tissue from patients with advanced BPH. This introduces a selection bias that precludes the characterization of dynamic changes in intercellular communication during early pathogenesis and the identification of targets for early intervention. In addition, current research has not established molecular subtypes of BPH based on intercellular communication profiles, nor has it comprehensively validated the correlation between these interaction features and patient clinical parameters (IPSS score, prostate volume, drug response, and prognosis). This fails to account for inter-patient heterogeneity, rendering these findings yet unable to guide precision clinical decision-making. The development of therapeutic targets targeting intercellular communication lags significantly behind; many predicted core pathways remain in the preclinical phase and have not entered clinical trials, hindering translation from the laboratory to the bedside. To address these limitations, future research must integrate single-cell and spatial multi-omics analysis, standardized analytical workflows, robust functional validation, and large-scale clinical cohorts studies. Only then can the findings from intercellular communication research be translated into precision treatment strategies for BPH, particularly for non-androgen-dependent targeted therapies.

## 4 Identification and translation of BPH therapeutic targets at single-cell resolution

**Figure 3:**
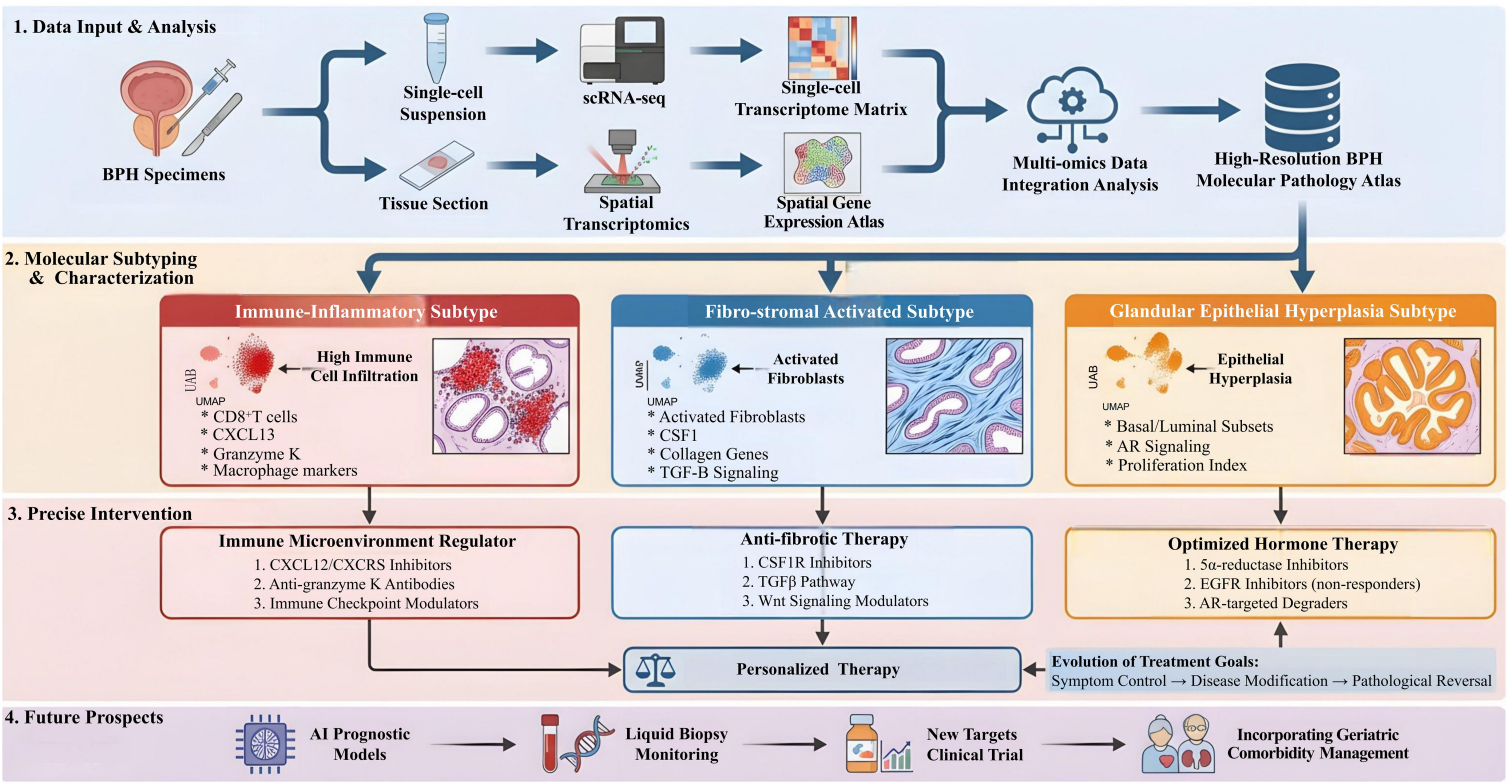
A precision medicine roadmap for BPH guided by single-cell molecular subtyping. Integrated scRNA-seq and spatial transcriptomics define three molecular subtypes: Immune-Inflammatory Subtype (high immune infiltration), Fibro-stromal Activated Subtype (fibrotic signature), and Glandular Epithelial Hyperplasia Subtype (AR-driven proliferation). Each subtype has its corresponding characteristics, such as high CD^+^8T cell infiltration and high CXCL13 expression in the immune inflammatory subtype; The Fibro-stromal Activated Subtype is characterized by activated fibroblasts; The Glandular Epithelial Hyperplastic Subtype is characterized by androgen receptor drive. And each subtype provides targeted treatment strategies based on its characteristics, which can be combined into personalized treatment plans aimed at changing the disease. The future direction is based on multi omics analysis, including single-cell sequencing, combined with artificial intelligence, to predict new therapeutic targets and multi-modal precision personalized therapy.

The heterogeneity of cellular architectures and pathogenic networks in BPH revealed by single-cell RNA sequencing fundamentally redefines traditional treatment strategies. To transition from symptom management to disease-modifying therapies, molecular insights at single-cell resolution must be translated into a precision medicine framework that informs clinical decision-making. As shown in **Figure 3**, this framework categorizes BPH into three distinct molecular subtypes: immune-inflammatory, fibro-stromal activated, and glandular epithelial hyperplasia. This classification enables the development of tailored interventions targeting specific pathways such as the CXCL12/CXCR5 or CSF1R axes. Beyond stratification, single-cell resolution identifies biomarkers that underpin resistance to conventional 5α-reductase inhibitors, including BE5 cells and TREM2+ macrophages. By leveraging spatial transcriptomics and AI-driven prognostic models, this systematic strategy facilitates the development of companion diagnostics, aiming to shift the clinical objective toward the fundamental reversal of pathological remodeling.

### 4.1 Mechanisms of heterogeneity in drug response

The cellular heterogeneity and non-androgen-driven pathways revealed by single-cell sequencing fundamentally explain the mechanisms of resistance to existing androgen-targeted therapies, offering new avenues for precision treatment. This chapter elucidates how single-cell sequencing identifies drug resistance mechanisms, discovers therapeutic targets, and addresses the challenges of large-volume BPH, thereby underscoring the clinical value of targeting non-androgenic pathways. Currently, BPH management comprises a comprehensive system including active surveillance, pharmacotherapy, and various minimally invasive and conventional surgical interventions. Although drug standard therapy based on α-adrenergic receptor blockers and 5α-reductase inhibitors remains the mainstream approach, it is ineffective against fibrotic remodeling and chronic inflammation, fails to reverse detrusor dysfunction, and carries side effects often affect treatment adherence (84, 85). Future strategies are moving toward enhanced precision, reduced invasiveness, and superior disease-modifying effects.” Current medications primarily focus on symptom control and the prevention of acute events, lacking true disease-modifying therapies capable of arresting the abnormal proliferation of prostate stem cells or regulating age-related metabolic disorders.

To investigate the mechanisms underlying suboptimal responses to finasteride in some BPH patients, Yanting Shen et al. Performed integrated scRNA-seq and spatial transcriptomics on tissue samples from treated and untreated individuals. The results revealed that finasteride treatment induces basal cell proliferation while reducing AR-mediated transcriptional activity and increasing luminal epithelial apoptosis, thereby compromising the epithelial barrier (49). This barrier dysfunction leads to the increased secretion of TGF-β into the periglandular space, which subsequently upregulated PCNA via EGFR activation, driving basal cell proliferation. This study identifies a potential non-AR-dependent therapeutic strategy—EGFR inhibition—for patients with basal cell-type BPH who are refractory to finasteride.

In the search for novel BPH therapeutics, Ming Zhan et al. utilized scRNA-seq to demonstrate that CSF1 secreted by prostate fibroblasts activates the PI3K/Akt signaling pathway via CSF1R, thereby promoting epithelial cell proliferation. Sunitinib was shown to pharmacologically inhibited this pathway and suppressed the progression of BPH (82). Additionally, research on resveratrol exemplifies the first integration of single-cell sequencing with network pharmacology: scRNA-seq was initially used to identify core non-androgenic targets(TGF-β1, IGF1, ITGA4) in BPH-activated fibroblasts and epithelial cells. Subsequently, network pharmacology was employed to construct a regulatory network linking resveratrol to these targets and pathways, revealing that resveratrol inhibition of the PI3K/Akt pathway (86), blocks non-androgen-dependent fibroblast activation and epithelial proliferation, ultimately mitigating BPH progression. This study demonstrates a refined approach to data-guided drug screening, overcoming the lack of cellular specificity often associated with traditional network pharmacology. While inflammatory cell infiltration is associated with prostate enlargement and symptom severity; standard medications lack targeted anti-inflammatory properties (54, 87, 88). Therefore, developing agents targeting underlying pathological mechanisms, including inflammation, fibrosis, and cellular senescence, is essential to address current therapeutic deficiencies and facilitate the transition from symptom management to disease-modifying therapies. The optimal model involves tailoring personalized treatment plans based on each patient’s pathological phenotype, such as proliferation pattern, inflammation, degree of fibrosis, and detrusor function.

### 4.2 Molecular characteristics and treatment strategies for large-volume BPH

Clinical symptoms and therapeutic interventions for BPH vary depending on prostate volume. For patients with predominantly dynamic obstruction, α-blockers rapidly improve symptoms by relaxing smooth muscle but are ineffective against glandular hyperplasia. For those with significant mechanical obstruction due to larger prostates, 5α-reductase inhibitors (such as finasteride) exert their effects by reducing gland volume, although their onset of action is slow. In cases of significantly enlarged glands where medical therapy fails to reverse marked anatomical obstruction, surgical resection remains the most definitive approach for relieving obstruction and preserving upper urinary tract function (89). To investigate the necessity of surgery for large-gland BPH, Nadia Atallah Lanman et al. performed scRNA-seq on tissue samples from large-volume (>90g) and small-volume (<40g) prostates. The results showed that non-polarized macrophages preferentially accumulate in large-volume BPH, with their abundance increasing in proportion to prostate volume. The Infiltration of lipid-rich macrophages may exacerbate LUTS in these patients, as their abundance correlates positively with body mass index and IPSS scores (71). Similarly, Meaghan M. Broman et al. also analyzed cellular heterogeneity in small- and large-volume BPH tissues via scRNA-seq, confirming that Taa cell in large-volume BPH secrete granzyme K to promote the secretion of SASP chemokines in fibroblast. This recruits immune cells and enhances the infiltration of aging-associated CD8+ T cells into the prostate (5), the intensity of which correlates positively with IPSS score. Therefore, scRNA-seq enables the identification of specific cell subpopulations and therapeutic targets associated with the pathogenesis of large-volume BPH, offering new avenue for drug innovation, particularly for patients refractory to conventional therapy.

### 4.3 Discovery of predictive biomarkers and therapeutic targets for BPH based on single-cell sequencing

This section outlines the translational value of scRNA-seq in precision medicine for BPH. The traditional BPH diagnostic and treatment system has two primary limitations: first, it relies solely on macroscopic clinical indicators such as IPSS scores and prostate volume, which fail to accurately predict disease progression or drug response; second, target development has traditionally centered on the androgen axis, leaving no effective intervention strategies for non-androgen-driven, drug-resistant BPH (8, 90, 91). In contrast, scRNA-seq, operates at single-cell resolution, overcoming the technical limitations of traditional bulk sequencing. It systematically identifies numerous cell-type-specific and non-androgen dependent prognostic biomarkers and therapeutic targets, providing essential evidence for the transition of BPH treatment from empirical models to precision medicine.

#### 4.3.1 Biomarkers for predicting drug response and treatment outcomes

Cell-specific biomarkers identified via scRNA-seq can be integrated with standard clinical indicators to enable precise stratification of BPH and prediction of treatment efficacy. For example, BE5 basal cells—identified in previous studies as the origin cells of BPH hyperplastic nodules—exhibit a positive correlation between their abundance and disease progression rate. When combined with baseline IPSS scores and prostate volume, these markers can identify populations at high risk for progression, thereby guiding early intervention for BPH (42). Similarly, the abundance of TREM2+ macrophages serves as a key inflammatory marker in large-volume BPH. When integrated with prostate volume and PSA levels, it can predict responsiveness to 5α-reductase inhibitors, suggesting that patients with high TREM2+ infiltration should transition from monotherapy to anti-inflammatory combination regimens (71). Furthermore, CXCL13 expression levels, which correlate with the senescence-associated cellular burden, can serve as a companion diagnostic biomarker to guide patient selection and evaluate the efficacy of senescence-targeted therapies (64).

To translate these scRNA-seq-derived candidate biomarkers into clinically actionable tools, it is essential to bridge the gap between high-dimensional single-cell profiling and routine diagnostic workflows. Given that scRNA-seq itself remains impractical for routine clinical use due to its high cost, requirement for fresh tissue dissociation, and extensive bioinformatics expertise, two complementary translational pipelines are warranted depending on the nature of the biomarker. For cell-subset markers such as BE5 basal cells and TREM2+ macrophages, which require *in situ* identification of specific cell populations within the tissue architecture, multiplex immunohistochemistry (mIHC) on formalin-fixed paraffin-embedded (FFPE) biopsy specimens represents the most feasible and cost-effective approach. mIHC enables simultaneous detection of multiple markers on a single tissue section, preserving spatial context and allowing quantification of target cell abundance relative to total cell populations. This technology is already widely available in routine pathology laboratories and can be readily integrated into existing diagnostic algorithms. In comparison, for soluble secreted factors such as CXCL13 and CSF1, liquid biopsy-based platforms—particularly enzyme-linked immunosorbent assay (ELISA) or chemiluminescence-based immunoassays using serum or urine samples—offer a minimally invasive, repeatable, and scalable detection strategy (92, 93). These assays can be performed on standard clinical laboratory equipment with rapid turnaround times, making them suitable for routine monitoring of treatment response and disease progression. Through this dual translational strategy incorporating both tissue-based (mIHC) and liquid-based (ELISA) assays, the molecular subtyping achieved at single-cell resolution can be effectively implemented as a clinically actionable detection system, facilitating the transition from empirical to precision-based management of BPH.

#### 4.3.2 Identification and translation of prognostic-related targets

Integrating the high-dimensional analytical power of scRNA-seq with computational tools enables the identification of potential prognostic biomarkers and therapeutic targets for BPH. Yichuan Wang et al. utilized scRNA-seq and Mendelian randomization analysis to identify six senescence-associated genes (MAP2K1, ATM, ATRAID, TP53, ITPR1, and SENP7) with putative causal relationships to BPH. Among these, MAP2K1, ATM, ATRAID, and TP53 were identified as protective factors for BPH, while ITPR1 and SENP7 were associated with an increased risk of BPH (94). The consistent expression patterns of these genes across diverse prostate cell types further support their functional association with BPH. Further analysis revealed that ATF3, EGR1, and FOS are the core transcription factors regulating these genes. In addition, scRNA-seq analysis of BPH tissue indicated that HSPA1A is significantly upregulated in both epithelial and stromal cells; it may inhibit apoptosis and oxidative stress via the ERK/JNK signaling pathway, thereby promoting BPH cell proliferation. Silencing HSPA1A increases apoptosis and reactive oxygen species (ROS), subsequently inhibiting BPH progression, while the HSPA1A antagonist KNK437 induces prostate atrophy—a functional validation of HSPA1A’s role in the pathogenesis of BPH (95). Furthermore, HSPA1A expression correlates positively with prostate volume, total prostate-specific antigen (tPSA), free prostate-specific antigen (fPSA), and IPSS. Overall, scRNA-seq serves as a robust reservoir of biomarkers and targets for non-androgen-targeted BPH therapies. Future translational research must leverage on single-cell molecular subtyping to achieve precise patient stratification, while simultaneously developing cell-specific targeted drugs and companion diagnostics of facilitate the shift from “symptom-based treatment” to “cause-based treatment.” In summary, these findings underscore the critical role of scRNA-seq in BPH research by enabling the prediction of prognostic biomarkers and the identification of new therapeutic targets, which holds significant implications for future clinical management.

## 5 Future prospects: toward a non-androgen-dependent paradigm

ScRNA-seq is redefining our understanding of BPH pathophysiology, providing a high-resolution microscopic landscape and paving the way for a fundamental shift from the traditional androgen-dependent model toward a strategy based on microenvironmental regulation and cellular heterogeneity. The realization of this shift will require breakthroughs and systematic integration across the following key areas:

### 5.1 Constructing a three-dimensional spatiotemporal molecular pathology atlas of BPH

While single-cell transcriptomics has systematically revealed the non-androgen-driven mechanisms of BPH, transcriptomic analysis alone remains insufficient to fully elucidate the multidimensional regulatory networks underlying disease onset. Future research must expand beyond the transcriptomic level; there is an urgent need to construct a three-dimensional spatiotemporal molecular pathology atlas of BPH through the integration of multiple technologies. Single-cell multi-omics technologies can simultaneously analyze transcriptomic, epigenomic, proteomic, and metabolomic features within the same cell, overcoming the limitations of single-transcriptome analysis (96). This approach will systematically map the regulatory cascade of non-androgen pathways across epigenetic regulation, protein translation, and metabolic reprogramming, thereby identifying the core nodes driving BPH progression. This atlas will not only identify cell subpopulations but also precisely localize them within the tissue, characterize their gene regulatory networks (driven by epigenetics), and reveal intercellular communication via signaling pathways. Spatial transcriptomics and spatial metabolomics preserve *in situ* spatial information, precisely resolving the local microenvironmental architecture within hyperplastic nodules in the BPH transition zone. They reconstruct the spatial distribution of non-androgen-dependent signaling loops and *in situ* cellular interaction patterns, elucidating the mechanisms of focal progression. An AI-powered analysis framework can address common challenges such as batch effect correction across cohorts, standardized annotation of stromal and immune subsets, and multidimensional target prediction, enhancing data integration and target discovery. Furthermore, integrating prostate organoids with single-cell sequencing enables the construction of *in vitro* models that recapitulate human BPH features, facilitating longitudinal tracking of disease progression and high-throughput drug screening (97). This provides a robust framework for target validation and drug development aligned with clinical practice.

### 5.2 A single-cell-guided precision medicine system for BPH

The cellular heterogeneity and non-androgen-driven mechanisms of BPH revealed by scRNA-seq must ultimately be translated into clinically actionable diagnostic and treatment strategies. Establishing a single-cell-guided precision medicine system is essential to address current limitations in BPH management. Based on molecular profiles, BPH can be classified into three core subtypes: immune-inflammatory-dominant, fibrotic-stromal-dominant, and glandular-epithelial-proliferation-specific. By correlating the molecular features of each subtype with clinical phenotypes, including IPSS scores, prostate volume, PSA levels, and response to 5α-reductase inhibitor therapy, clinicians can select the optimal treatment regimen, thereby achieving personalized care. However, implementing this system depends on mapping complex molecular subtypes onto standard diagnostic dimensions. Based on integrated single-cell data and clinical cohort, we propose the following three core molecular subtypes and their clinical integration strategies. 1. Immune-inflammatory subtype: Characterized by increased infiltration of TREM2+ and MARCO+ macrophages and CD8^+^ T cells, alongside upregulated expression of CXCL13/CXCL5. For such patients, if response to 5α-reductase inhibitor monotherapy is suboptimal, combination therapy with anti-inflammatory agents or CSF1R pathway inhibitors may be considered (64, 71). 2. Fibrosis- stromal subtype: Characterized by pronounced activation of fibroblasts(particularly pericentral fibroblasts), enrichment of TGF-β and FGF signaling pathways, and increased extracellular matrix deposition. Such patients may show limited response to conventional monotherapies; anti-fibrotic agents targeting TGF-β signaling or mechanotransduction pathways may be more effective (49, 82). 3. Glandular-epithelial subtype (androgen-independent): Characterized by enrichment of the BE5 basal cell subset, activation of Wnt and EMT pathways, exhibiting relative independence from AR signaling (42, 43). For these patients, novel therapies targeting the FGF or Wnt pathways, or those facilitating senescent cell clearance, should be prioritized.

Future drug development will focus on modulating the abnormal microenvironment. For example, this may involve suppressing chronic inflammation by regulating the recruitment or polarization of specific immune cells (such as regulatory T cells); developing anti-fibrotic therapies to reverse stromal hardening; or targeting abnormal paracrine signaling pathways that drive proliferation. Concurrently, single-cell sequencing can guide the development of non-androgen-dependent targeted drugs and the optimization of combination therapy regimens with conventional pharmacotherapy. This will enable tailored strategies for different subtypes that synergistically block both androgen pathways and core non-androgen pathogenic axes, thereby improving outcomes for refractory BPH. However, key bottlenecks in clinical translation (including the lack of non-invasive diagnostic systems, poor integration of molecular subtyping with routine clinical workflows, and lengthy clinical validation cycles for therapeutic targets) must be addressed to accelerate the translation from bench to bedside.

### 5.3 Challenges and prospects

ScRNA-seq and single-cell multi-omics technologies have significantly refined our understanding of BPH pathology with unprecedented resolution, shifting the focus beyond the traditional view that prostatic hyperplasia is driven solely by androgens. These technologies demonstrate that BPH is a dynamic pathological process characterized by extensive cellular and molecular heterogeneity, in which non-androgen-dependent mechanisms (such as the inflammatory microenvironment and EMT) may play a role comparable in significant to the classic androgen pathway. While providing a framework for elucidating non-androgen-driven mechanisms in BPH and establishing precision medicine systems, current research still faces field-specific general technical limitations Addressing these challenges remains a primary focus for future investigation.

Currently, scRNA-seq applications in BPH research face several critical bottlenecks. First, existing studies rely heavily on end-stage surgical specimens; whereas early-stage clinical samples are difficult to acquire. Furthermore, most analyses are single-center, cross-sectional studies with limited sample sizes, lacking dynamic tracking within large-scale longitudinal cohorts. This precludes the elucidation of early molecular events, the mechanisms triggering non-androgenic pathways, and the dynamic patterns of disease progression. Second, fundamental anatomical and cellular divergences exist between human and mouse prostates. Existing animal models fail to recapitulate the pathological features of human BPH, hindering the translation of basic research into clinical applications; Third, t research remains insufficient regarding the region-specific mechanisms of the prostate, particularly the cellular and molecular basis for the predisposition of the transition zone toward hyperplasia. Fourth, a standardized molecular classification system for BPH with clinical utility has not yet been established, making limiting support for precise patient stratification and personalized interventions.

Furthermore, BPH tissue is characterized by a dense stroma, making single-cell dissociation significantly more challenging than in other solid tissues. Inconsistencies in tissue processing, data analysis, and cell annotation standards across existing studies result in limited reproducibility and poor data integrability. Meanwhile, the high cost and technical barriers associated with clinical-grade single-cell testing limit their large-scale implementation and broader clinical translation.

In the future, as technological platforms mature and multidisciplinary collaboration deepens, single-cell technology will drive BPH research beyond descriptive cellular atlases toward a full-chain development encompassing mechanistic dissection and clinical translation, thereby fundamentally reshaping the diagnostic and therapeutic landscape for BPH. At the mechanistic level, research will shift from unidimensional transcriptomic analysis to integrated studies of single-cell multi-omics and spatial transcriptomics, constructing a multidimensional “cellular-molecular-spatial” pathology atlas. This will systematically elucidate the regulatory networks of core non-androgenic pathways—including SASP, local metabolic reprogramming, abnormal innervation, and immune/stroma/epithelial interactions, as well as the crosstalk with androgen pathways. Such insights will delineate the multifactorial nature of pathological remodeling in BPH and address knowledge gaps regarding the region-specific and longitudinal evolution of the disease. At the clinical translation level, this research will facilitate the transition from traditional androgen-dependent symptomatic management toward precision medicine targeting non-androgen-driven mechanisms. By establishing a standardized molecular classification system for BPH based on large-scale single-cell data, we will enable precise patient stratification, risk assessment, and personalized therapy selection. Simultaneously, the therapeutic focus will expand beyond hormonal inhibition and smooth muscle relaxation toward etiological interventions, such as immune microenvironment modulation, anti-fibrotic therapy, and metabolic resetting, thereby achieving a fundamental leap from “glandular reduction” to “restoration of tissue homeostasis”.

Although single-cell research on BPH still faces challenges such as tissue accessibility, translation barriers, and technical standardization, this technology has fundamentally transformed our understanding of the disease, establishing new frontiers for research into non-androgen-dependent mechanisms and precision therapeutics. In the future, through multidisciplinary synergy, the establishment of standardized protocols, and the standardized protocols, single-cell technology will undoubtedly reshape the BPH management system, providing patients with safer, more effective, and sustainable personalized treatment options.
